# Epigenetics and Inflammatory Markers: A Systematic Review of the Current Evidence

**DOI:** 10.1155/2019/6273680

**Published:** 2019-05-08

**Authors:** Valentina Gonzalez-Jaramillo, Eliana Portilla-Fernandez, Marija Glisic, Trudy Voortman, Mohsen Ghanbari, Wichor Bramer, Rajiv Chowdhury, Tamar Nijsten, Abbas Dehghan, Oscar H. Franco, Jana Nano

**Affiliations:** ^1^Department of Epidemiology, Erasmus MC, Erasmus University Medical Center, 3015 CN, Rotterdam, Netherlands; ^2^Institute of Social and Preventive Medicine (ISPM), University of Bern, 3012, Bern, Switzerland; ^3^Division of Vascular Medicine and Pharmacology, Department of Internal Medicine, Erasmus University Medical Center, 3015 CN, Rotterdam, Netherlands; ^4^Leibniz Institute for Prevention Research and Epidemiology-BIPS, Bremen, Germany; ^5^Department of Genetics, School of Medicine, Mashhad University of Medical Sciences, 13131, Mashhad, Iran; ^6^Medical Library, Erasmus MC, Erasmus University Medical Center, 3015 CN, Rotterdam, Netherlands; ^7^Department of Public Health and Primary Care, University of Cambridge, Strangeways Research Laboratory, CB1 8RN, Cambridge, UK; ^8^Department of Dermatology, Erasmus MC, Erasmus University Medical Center, 3015 CN, Rotterdam, Netherlands; ^9^Department of Biostatistics and Epidemiology, MRC-PHE Centre for Environment and Health, School of Public Health, Imperial College London, W2 1PG, London, UK; ^10^Helmholtz Zentrum München, German Research Center for Environmental Health, Institute of Epidemiology, D-85764, Neuherberg, Germany; ^11^German Center for Diabetes Research (DZD), D-85764, Munich-Neuherberg, Germany

## Abstract

Epigenetic mechanisms have been suggested to play a role in the genetic regulation of pathways related to inflammation. Therefore, we aimed to systematically review studies investigating the association between DNA methylation and histone modifications with circulatory inflammation markers in blood. Five bibliographic databases were screened until 21 November of 2017. We included studies conducted on humans that examined the association between epigenetic marks (DNA methylation and/or histone modifications) and a comprehensive list of inflammatory markers. Of the 3,759 identified references, 24 articles were included, involving, 17,399 individuals. There was suggestive evidence for global hypomethylation but better-quality studies in the future have to confirm this. Epigenome-wide association studies (EWAS) (n=7) reported most of the identified differentially methylated genes to be hypomethylated in inflammatory processes. Candidate genes studies reported 18 differentially methylated genes related to several circulatory inflammation markers. There was no overlap in the methylated sites investigated in candidate gene studies and EWAS, except for* TMEM49, *which was found to be hypomethylated with higher inflammatory markers in both types of studies. The relation between histone modifications and inflammatory markers was assessed by one study only. This review supports an association between epigenetic marks and inflammation, suggesting hypomethylation of the genome. Important gaps in the quality of studies were reported such as inadequate sample size, lack of adjustment for relevant confounders, and failure to replicate the findings. While most of the studies have been focused on C-reactive protein, further efforts should investigate other inflammatory markers.

## 1. Introduction

Inflammation is a critical response to pathogens and injuries in the human body. Specifically, chronic low-grade inflammation plays a key role in the pathogenesis of chronic conditions and diseases like obesity, diabetes mellitus, and cardiovascular disease [1–3]. A better understanding of factors that contribute to the development of inflammation and its consequences on disease is essential to improve prevention strategies in inflammation-related disorders.

Genome-wide association studies have identified several genetic variants associated with inflammatory markers such as C-reactive protein, the most widely studied marker [4, 5], but the explained variance is relatively small. In addition, nongenetic factors such as smoking and dietary behaviours have been shown to exhibit a strong influence on the inflammatory response [6, 7]. Emerging evidence suggests that epigenetic processes, reflecting changes in gene expression that occur without sequence mutations, may offer opportunities to understand the pathophysiology of inflammation processes. The role of epigenetic determinants is increasingly being recognized as a link between environmental factors and disease risk. Moreover, epigenetic modifications are also involved in differentiation of the immune cells, a key component of the inflammatory process. Epigenetics is defined as a group of chemical modifications of the DNA sequence, which could be affected by external factors such as BMI, smoking, and inflammation and can be transmitted from one generation of cells to the others [8]. The molecular basis of epigenetic mechanisms is complex and comprises DNA methylation, modifications of histones, and gene regulation by noncoding RNAs [9]. Unlike genetic variation, epigenetic modifications are dynamic and potentially reversible and, therefore, could be modified by lifestyle and other therapeutic approaches.

Until now, a comprehensive and systematic appraisal of the current literature on the role of epigenetic modifications in inflammation measured by levels of inflammatory markers is missing. Therefore, we aimed to identify and synthetize all available evidence conducted in humans and quantify the association of two of the major epigenetic modifications, DNA methylation, and histone modifications, with circulation inflammatory markers in blood.

## 2. Materials and Methods

This review was conducted and reported using a predefined protocol and in accordance with the PRISMA [10] and MOOSE [11] guidelines (Supplement Material S1 and S2). We sought studies published before 21 November of 2017 (date last searched) in five electronic databases: Embase.com, Medline (Ovid), Web-of-Science, Cochrane Central, and Google Scholar. We did the search with the help of an experienced medical information specialist. In databases where a thesaurus was available (Embase and Medline), articles were searched by thesaurus terms, title and/or abstract and in other databases, only by title and/or abstract. The search combined terms related to the exposure (e.g., epigenetic, hypomethylation, hypermethylation, DNA methylation, and histone acetylation) and outcome (e.g., inflammation, C-reactive protein, and cytokine). We did not apply any language restriction, but we restricted the search to studies on humans alive. The full search strategies of all databases are provided in Supplement Material S3. The study identification also included manual search, based on the screening of the citations of the relevant studies.

Information about study selection and inclusion criteria, data extraction process, and risk of bias assessment is described in Supplement Material S4.

## 3. Results

**Figure 1:**
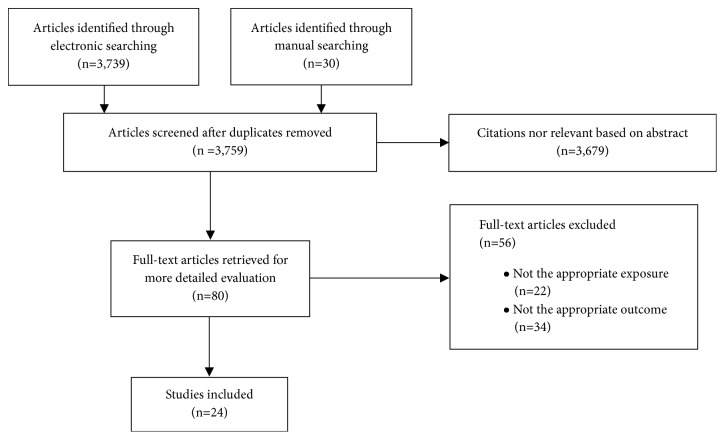
Flowchart of studies included in the systematic review.

After deduplication, we identified 3,759 potentially relevant citations. Based on the title and abstract, 3,679 studies were excluded due to inappropriate exposure (gene mutations, gene polymorphism, and microRNA), inappropriate outcome (autoimmune diseases, cancer, and inflammation-related diseases such as asthma), or both. We also excluded investigations conducted in mice or rats (n=298). Additionally, we excluded studies that reported methylation levels in inflamed body areas or inflamed cells without quantitative investigation with inflammation markers, as well as studies that assessed methylation changes before and after immunotherapy. The final set of 80 studies were considered for full-text assessment. Of these, 24 unique studies met our eligibility criteria and were included in this review. The other 56 articles were excluded for reasons shown in Figure 1.

### 3.1. Characteristics of the Included Studies

Detailed characteristics of the included studies are summarized in Tables 1–3. All included studies were of cross-sectional design, except one study of prospective design [12]. Overall, 17,399 individuals were participating in these studies. Nine studies included participants from the USA, three studies from China, three studies from Canada, and the rest included participants from Brazil, Colombia, India, Ireland, Germany, Greece, Mexico, Spain, and Sweden. One of the studies [13] included participants from different cohorts such as USA, UK, Italy, Germany, and Netherlands. The majority (n=23) of studies assessed epigenetic signatures in blood, whereas other assessed epigenetic marks in tumour specimens (glioblastomas).

Of the 24 studies included, four studies assessed only global DNA methylation, eleven studies assessed only DNA methylation in specific candidate genes, and seven studies used genome-wide approaches. One additional study examined both global DNA methylation and methylation in specific candidate genes [14]. Only one study assessed histone modification in relation to inflammation markers [15].

The most studied marker was C-reactive protein (CRP), which was evaluated in 17 studies. Interleukins like IL-4, IL-6, IL-8, IL-9, IL-10, and IL-18 were evaluated in 11 studies. TNF-*α* was assessed in three studies, fibrinogen was assessed in two studies, and other markers such as VCAM, ICAM, VEFG, COX2, leptin, TNFR2, C-CAM1, alpha interferon, and TGF-*β* were assessed one single time. Nine studies were judged at medium risk of bias whereas the rest were at high risk of bias.

### 3.2. Global DNA Methylation and Inflammatory Markers

Five studies examined the association between global DNA methylation and inflammatory markers in blood samples (Table 1). Four of these studies assessed methylation in long-interspersed nuclear element (LINE-1). A large portion of methylation sites within the genome are found in these repeat sequences and transposable elements and correlate well with total genomic methylation content. From the four studies, two [14, 16] reported no association between global DNA methylation and CRP levels, while the other two showed lower methylation to be related with higher CRP levels [17, 18].

One study [16], in addition to CRP levels, also evaluated the association of global DNA methylation at LINE-1 with VCAM-1 and ICAM-1 and reported an inverse association with VCAM-1 but no association with ICAM-1. One study quantified global DNA methylation by measuring the amount of methylated cytokines in the sample (5 mc) relative to global cytidine (5mC + dC) in a positive control and found no association between global DNA methylation and IL-6 serum levels [19].

### 3.3. Gene-Specific DNA Methylation and Inflammatory Markers

Twelve studies examined the relation of inflammatory markers with methylation sites in, or near, candidate genes (Table 2). One study measured DNA methylation in tumour specimens [20], whereas the other studies used blood samples to assess the DNA methylation.

Of the twelve studies, eight did not report any level of adjustment or control for confounders, one of them controlled for age and sex [21], and the others controlled for these two confounders plus additional ones such as diet and race [14, 22, 23]. Of the twelve studies, three focused solely on CRP as outcome, one solely on interleukins, and one solely on leptin and the others assessed a set of inflammatory markers including interleukins, TNF-*α*, and fibrinogen.

In total, eight studies assessed CRP as inflammatory marker. Overall, these studies found higher levels of CRP to be associated with higher degree of methylation of* SOCS-1* [24],* LY86 *[22], and* EEF2* [25] and higher levels of CRP to be associated with lower degree of methylation of* AIM2 [26]*,* IL-6* [27], and* IL-6 promoter *gene [21]. One additional study that examined methylation levels of* IL-6* promoter and CRP reported no association [14]. In addition, no association was found between methylation status of* F2RL3* in peripheral blood cells and CRP levels.

Five studies evaluated the association of gene-specific DNA methylation with* IL-6*. They found higher degree of methylation of* MGMT, RARβ, RASSF1A, *and* CDH13* in tumour specimens and of* SOCS-1* in peripheral blood with higher levels of* IL-6*, while others found less degree of methylation of* USP2, TMEM49, SMAD3, DTNB*, and* IL-6* promoter with higher levels of IL-6. Other interleukins such as IL-8, IL-10, and IL-18 were only evaluated once [20, 23, 28]. No significant correlation was found for IL-8, whereas for IL-10 and IL-18 inverse association was found with DNA methylation in* IL-10* promoter and* F2RL3*, respectively (Table 2).

Two studies evaluated leptin as outcome, showing contradictory results. One reported inverse association between leptin levels and* Leptin Receptor* methylation [25], whereas the other reported no association between* Leptin promoter *and leptin levels [29].

Two studies assessed the association of DNA methylation and TNF*α* levels. Higher levels of methylation of* EEF2* [25] and* SOCS-1* [24] were found with higher levels of TNF*α*.

Additionally, six studies reported the association between methylation at different genes (*MGMT, RARβ, RASSF1A, CDH13, USP2, TMEM49, EEF2, COL18A1, IL4I1, LEPR, PLAGL1, IFRD1, MAPKAPK2, PPARGC1B, SMAD3, DTNB, LY86,* and* F2RL3*) with levels of several inflammatory markers other than CRP and interleukins (VEGF, VCAM1, C-CAM1, COX-2, sTNFR2, and fibrinogen) (Supplement Table 1).

### 3.4. Epigenome-Wide Analysis and Inflammatory Markers

Seven studies investigated differentially methylated regions in the genome in a hypothesis-free approach. Six of them adjusted at least for age and sex. Of these six, four adjusted additionally for BMI, smoking, and/or other confounders. All of the studies used blood samples to assess DNA methylation.

One study assessed 121 biomarkers related with inflammation, cancer, and cardiovascular disease [30] and five studies assessed CRP. The remaining two studies evaluated TNF and interleukins such as IL-1*β*, IL-6, IL-8, and IL-10 [31, 32] (Table 3).

Three out of seven studies used replication to validate their findings: two of them [13, 33] used external validations and one [34] internal validation.

The identified genes were enriched by pathways such as atherosclerosis, IL-6, IL-9, IL-8, growth hormone, and JAK/STAT signalling pathways.

Among the genes reported to be differentially methylated,* SOCS3* and* BCL3 *were found to be significantly hypomethylated in two studies [13, 33].* BCL3* was no longer significant in the replication cohort, whereas* SOCS3 *remained significant after replication.

### 3.5. Histone Modifications and Inflammatory Markers

Only one study examined the association between histone modifications and inflammatory markers [15]. The authors assessed levels of acetylated histone H4 in the peripheral blood mononuclear cells of Chronic Obstructive Pulmonary Disease (COPD) patients and reported higher acetylation levels in patients with higher IL-8 levels and in patients with lower IL-4 levels.

## 4. Discussion

This is the first attempt to summarize current literature on the role of epigenetic marks in chronic inflammation. There is suggestive evidence for hypomethylation of overall genome in inflammatory processes, but better-quality studies have to confirm these results. Histone modification and inflammatory markers are scarcely investigated. Given the complexity and variability of proteins involved in the inflammation network, most of the studies focused on exploring CRP levels with few studies on IL-6 and fewer investigations on IL-8, IL-10, IL-18, VEGF, Cox-2, TNF-*α*, sTNFR2, leptin, and fibrinogen levels. The largest epigenome-wide association study up to date found* AIM2* and* SOCS3* to be top genes related to CRP levels in whole blood.

### 4.1. Global DNA Methylation

There were either no or an inverse association of inflammatory markers such as CRP, VCAM-1, and ICAM-1 in whole blood. Because we identified only a small number of studies, we cannot make any firm inferences on the overall hypomethylation of the genome due to inflammation. Moreover, populations were hardly comparable as two of the studies were conducted on children while the others on adults. As global DNA hypomethylation has become the hallmark of most human cancers, stroke, and heart disease [35–38], the need to measure this epigenetic signature has become more essential. Global methylation would enable the ability to associate, for example, LINE-1 or 5-mdC levels, with correlative factors such as patient history or clinical outcome. The observed hypomethylation could lead to activation of dormant repeat elements and the subsequent aberrant expression of associated genes or may contribute to genomic instability and increased mutation rates. More intense efforts in studies investigating global DNA methylation through different methodologies such as Alu repeats and LUMA can hold future prospects for guiding risk stratification in individuals with high levels of inflammatory markers at an increased risk of chronic diseases.

### 4.2. EWAS vs. Candidate Gene Approaches

Ligthart et al. identified and validated 58 CpG sites located in 45 unique loci in whole blood among 12,974 individuals of European and African descent [13]. The top signal near* AIM2* gene was found to be inversely associated with gene expression levels and with lower CRP levels.* AIM2* is a key regulator of human innate immune response implicated in defence mechanism against bacterial and viral pathogens [39, 40]. Several of these hits including cg18181703 (*SOCS3*), cg06126421 (*TUBB*), and cg05575921 (*AHRR*) were associated with future incidence of coronary heart disease and smoking [13], whereas two other CpGs were recently identified in an EWAS of type 2 diabetes [41]. The gene* SOCS3*, suppressor of cytokine signalling 3, plays a pivotal role in the innate immune system as a regulator of cytokine signalling along the JAK/STAT pathway and was previously reported to have an important role in the processes of atherosclerosis [42]. Moreover, another epigenome-wide association study conducted among 1,741 individuals of European descent reported* SOCS3*, among others, to be significantly associated with systemic CRP levels, not only in peripheral blood tissue, but also in human liver tissue [33].

Given the reported association of CRP levels and these cardiometabolic clinical outcomes, it seems that inflammation-related epigenetic features may explain part of the observed associations reported in epidemiology. However, the results should be interpreted with caution, as the association of CRP and DNA methylation were not adjusted for these factors. Most of the replicated CpG sites reported in the study of Lighart et al. were associated with different cardiometabolic phenotypes (body mass index, fasting glucose, fasting insulin, triglycerides, total cholesterol, and HDL-cholesterol), highlighting the evidence of a pleiotropic network of epigenetics across various phenotypes. This information is promising as it holds new insights into shared epigenetic mechanisms and provides opportunities to link the inflammation processes with clinical outcomes. Moreover, two large cohorts (KORA and GENOA) observed hypomethylation to be related with higher levels of CRP [33, 43]. The latter, reported a similar trend of hypomethylation among individuals of older age and suggested that these patterns of modifications of DNA methylation on CpG islands between aging and inflammatory markers may indicate shared molecular mechanisms underlying chronic diseases through epigenetic changes [43].

Differentially methylated genes associated with CRP levels and other inflammatory markers did not directly overlap with the genes identified from previously reported genome-wide association studies influencing CRP levels and other biomarkers. The nonoverlap between GWAS and EWAS identified genes shows that clinical phenotypes are being influenced by different molecular mechanism, all of them important to explain phenotypical variation. Most of the identified genes are involved in common inflammation pathways related to cancer, rheumatic diseases, and gastrointestinal pathologies [24, 27]. Nevertheless, candidate gene approaches have less stringent criteria to assign significance on the expense of a narrower focus on genes. This might explain the absence of reproducibility of results in the reviewed epigenome-wide association studies, except for* TMEM49*, which was found to be inversely associated with sTNFR2 and IL-6 levels in the candidate gene approach study of Smith et al, and shared the same direction of association with CRP levels, in the EWAS study of Lighart et al.

### 4.3. Histone Modification

This review demonstrated that evidence involving inflammation and histone modification mechanisms are inexistent. Histone modifications are another epigenetic mark that play a pivotal role in the epigenetic regulation of transcription and other functions in cells. In addition, histone modifications have been linked to other inflammatory-related disorders, such as dyslipidaemia, obesity, diabetes, cancer, and cardiovascular disease [44–46]. Future studies on histone modifications and inflammation markers might shed light on their functional role in chronic diseases and might provide novel target therapies for inflammatory conditions.

### 4.4. Bias, Confounding, and Tissue Specificity

There is quite ample evidence showing differential DNA methylation differing by ethnicity [47]. Therefore, it is recommended that studies investigating epigenetics of genes related to inflammation should replicate their findings in diverse populations. The largest to date epigenome-wide association study investigating DNA methylation and CRP levels used as discovery set a large European population (n = 8,863) and sought transethnic replication in African Americans (n = 4,111) [13]. As in genetic studies, the importance of replication of the significant findings in epigenetic association studies is a paramount in order to prevent false-positive results [48, 49].

Unlike genetic association studies that are less prone to confounding, epigenetic signatures throughout the genome are highly labile due to temporal or spatial factors affecting DNA such as age, gender, demographics, lifestyle, comorbidities, and medication use. It has been shown that methylation investigations harbour new information in explaining the variation of complex traits such as inflammation characterized by a strong influence of environment [4, 13, 50]. Inflammatory markers such as CRP, one of the most studied, are affected by both genetic and environmental factors. Therefore, controlling for confounders in epigenetic studies is crucial. In our review, the majority of our studies (62.5%) were classified as low quality largely explained by the lack of proper adjustment in the statistical models. While epigenome-wide studies controlled for life-style factors such as smoking, alcohol consumption, and BMI, candidate-gene approach studies were heavily suffering from incomplete adjustments.

Most of the inflammatory markers, and especially the ones of the acute phase, are predominantly synthesized in liver cells and hepatocytes and are regulated via transcription factors such as STAT3, C/EBP family members, and NF-kappa B by the proinflammatory cytokines IL-6 and IL-1*β* [51, 52]. Nevertheless, extrahepatic expression to a lesser degree has been reported for adipose tissue and blood cells [52]. DNA methylation profiles have been commonly studied in whole blood due to the easy access to the biological samples. Environmental exposure signatures such as smoking, alcohol, and other conditions involving the circulatory system and the immune response are well reflected in whole blood. This tissue is primarily composed of leukocytes, a key component of the human immune system and, therefore, highly relevant to systematic inflammation. However, since peripheral blood constitutes a heterogeneous admixture of different cell populations, it is possible that the results reflect inflammation-related DNA methylation changes that influence a single cell type component of blood cells. Adjusting for measured or estimated blood cell proportions, or future studies conducted in cell specific tissues, would help to rule out presence of any residual confounding caused by white blood cell distribution.

### 4.5. Causality and Study Designs

In the last years, the GWAS have resulted in the identification of many genetic variants that are associated with clinical traits and diseases. However, together, these variants explain only a small fraction of the variability. It has been suggested that epigenetics might hold promise to uncover the rest of the missing heritability. Moreover, it has been commonly hypothesized that epigenetic signatures are a cause for disease, rather than consequence. With the current evidence, it is unclear if epigenetic variation is causal to these inflammatory markers. In a recent study of Ahsan et al, the authors investigated the genetic and epigenetic influence in a large set of disease-related inflammatory markers [30]. Combining results of GWAS/EWAS in around 1,069 individuals and employing a complex bidirectional model to asses causality between genetic variation-DNA methylation-inflammation markers, it was concluded that DNA methylation has a limited direct effect on inflammatory markers. It reflects the underlying pattern of genetic variants, environmental exposures or secondary effect of the disease pathogenesis. In line with recent evidence, rather than a cause, DNA methylation seems to be a consequence of clinical traits, such as BMI [53].

All of the included studies in this systematic review were of cross-sectional design, except for one [47], meaning that both epigenetic signatures and outcomes were measured at the same time. This design challenges further inferences concerning causal relation, a typical vulnerability of epigenetic studies. In longitudinal cohort designs, repeated measurements for both inflammatory markers and dynamic methylation changes could improve our knowledge of the directionality of events. Furthermore, statistical approaches like Mendelian Randomization, in which genetic variants are used as proxies for DNA methylation and the outcome of interest, offer new opportunity to investigate the directionality of evidence from cross-sectional data [54]. The identification, directionality, and molecular pathways underlying the relation between epigenetic signatures and inflammatory markers represent promising targets for future functional studies.

### 4.6. Epigenetic Screening

In the last years, many advances in technologies related to measurements of epigenetic signatures have been developed to respond to the fast-growing pace of the field [55]. These techniques allow the investigation of DNA methylation either on candidate genes or on the whole-genome level. However, as the number of genes of interest increases along with the number of tissues of relevance, investigating the role of DNA methylation in different clinical traits could be very costly and time consuming. Progressing to more cost-effective solutions, high-throughput technologies have open new opportunities for epigenome-wide investigations in large-scale screening such as in population-based cohort studies. Furthermore, gene-specific assays such as bisulfite conversion provide a quick and efficient result for epigenetic investigations requiring relatively low DNA input with minimum DNA loss [56, 57]. Cloning, the gold standard method for gene-specific DNA methylation studies, followed by Sanger sequencing is another technological option [58]. Although the time for the procedure has been significantly reduced, the sequencing step might introduce several sources of errors [55, 59]. Another technique, pyrosequencing, represents a high throughput quantitative method used for bisulfite sequencing [60, 61]. This technique, which can be used for both DNA methylation and genetic variation (single nucleotide polymorphism) analysis, takes less time than cloning providing accurate reads within each run. Yet, optimal DNA quality is important to avoid misreads of pyrosequencing [55]. Mass spectrometry assay, on the other side, is a tool that can be used for the discovery and quantification of DNA methylation sites based on difference in fragments weights that have been cleaved depending on the methylation status [59]. This technology is highly sensitive and has the ability to sequence reads up to 600 bp, which is considerably longer than other methods. Quantitative Polymerase Chain Reaction (qPCR) arrays are another alternative of methylation quantification techniques operating on fluorophore-labelled probes that emit fluorescence when bound to a complementary DNA sequence. This method might not be ideal for regions with multiple CpG sites because many probes need to be created, resulting rather costly. However, if a region is characterized by a few CpGs, qPCR method might provide a simple and relatively inexpensive way to conclude a high-powered study [55].

Other chip techniques for epigenetic studies, in particular for histone modifications, include chromatin immunoprecipitation (ChIP), methylated DNA immunoprecipitation (MeDIP) platforms, and methyl-binding protein immunoprecipitation platforms. A major limitation to these techniques in epigenome-wide analysis is the quality of the antibody, which plays an important role in the proper enrichment of DNA. In general, the immunoprecipitation techniques require the availability of large sample volumes and only measure relative enrichment of epigenetic markers.

Concerning large-scale epigenetic analysis, the most widely used platforms, as shown from our review, are from Illumina. Illumina methylation profiling is based on bisulfite converted DNA genotyping [62]. For example, The Illumina Infinium HumanMethylation27 (27,000 CpG site) and Human-Methylation450 Bead (450,000 CpG sites) arrays provide genome-wide coverage, featuring methylation status at CpG islands, CpG shores, non-CpG sites, promoter regions, 5′ UTR, 3′ UTR, and gene bodies. More recent platforms, such as Infinium MethylationEPIC BeadChip Kit, have increased the number of interrogated sites to more than 850,000 CpGs across the genome at single-nucleotide resolution for only of 250 ng DNA as input quantity [63]. Moreover, TruSeq Methyl Capture EPIC Library Prep Kit, is another option that combines whole-genome bisulfite sequencing with methylation arrays that can support both screening and biomarker discovery studies targeting over 3.3 million CpGs [64]. These technologies rapidly produce a large amount of data at relatively low costs and are mostly preferred in population studies. On the other hand, epigenome-wide sequencing is another technology that is holding high hopes for future discoveries in the field of epigenetics. Currently, its widespread use is hampered by the high costs and computation burden of the analysis.

### 4.7. Clinical Implications

Understanding the epigenomic regulation of loci related to inflammatory markers might hold the possibility of discovering attractive targets for controlling inflammatory processes and, consequently, improving therapeutic interventions for chronic diseases that share in their aetiology, inflammatory-related pathophysiology. The identified epigenetic patterns may be used not only in functional studies to provide further insights into molecular mechanisms of inflammatory processes but also in biomarker studies using whole blood to improve the prediction of inflammation-related clinical disorders or events.

## 5. Conclusions

Current evidence suggests a potential role of epigenetics on the level of inflammatory markers in blood. Studies reporting on the association of inflammation with global DNA methylation show a hypomethylation trend. However, this evidence is not conclusive. Further studies are recommended to explore this relation. Moreover, studies on the role of histone modifications in inflammation markers are scarce. While most of the studies have been focused on CRP, reporting replicated genes across cohorts such as* SOCS3*, further efforts should focus on other biomarkers of the inflammatory cascade such as interleukins. Most importantly, given the systemic nature of inflammation, validation of the methylation sites among different tissues is paramount. The identified and reported genes so far involve epigenetics of inflammation with cardiometabolic factors, but also cancer and rheumatic diseases highlighting the potential of these regions as translational targets in the future. Given that we observed a lack of high quality investigations included in this review, we recommend future studies to improve some of the most urging factors such as an appropriate study design. This might be done by involving repeated measurements or with a prospective design that would allow drawing insights on one of the most important drawbacks of epigenetic data, assessing the directionality of the effects. Another important aspect to improve is to increase the sample size in order to provide adequate power and to perform proper adjustment of analysis to account for the role of environment on both epigenetics and inflammation. Lastly, the identified genes need to be validated in functional (in vitro and in vivo) studies in order to draw valuable and conclusive insights into the epigenetic mechanisms of inflammatory markers.

## Figures and Tables

**Table 1:** Global DNA methylation and inflammatory markers.

Author, Year	Study design	Outcome	Tissue type	Population Sex/Age/Sample size/Country	Adjustment	Main findings
*LINE-1 methylation*						

Baccarelli et al., 2010 [16]	CS	VCAM-1, ICAM-1 and CRP	WB	M /73.8 ± 6.7/n=593/USA	Age, BMI, smoking, pack years of smoking, IHD or stroke.	Inverse association for VCAM-1. No association for ICAM-1 and CRP.

Perng et al., 2012 [17]	CS	CRP	WBC	M and W/ 8.8 ± 1.7/n=568/ Colombia	Sex, vitamin A, maternal BMI and household socioeconomic stratum.	Higher CRP was related to lower LINE-1 methylation.

Zhang et al., 2012 [14]	CS	CRP	WBC	M and W/ 18-78/n=165/USA		No association (*β* coefficient=-0.02, p=0.81).

Narayan & Dangi, 2017 [18]	CS	CRP	WB	M and W/7.9 ± 1.5/n=600/India	Sex, plasma Vitamin A, socioeconomic status	Global DNA methylation was inversely related to CRP concentrations and the association was stronger in male children.

*5mdC*						

Murphy et al., 2015 [19]	CS	IL-6 (protein and serum levels)	WB	M and W/mean=33.04/ n=47/Ireland		No association (*r* = -0.125, p=0.46).

CS: cross-sectional; VCAM-1: vascular cell adhesion molecule 1; ICAM-1: intercellular adhesion molecule 1; CRP: C-Reactive protein; WB: whole blood; M: men; BMI: body mass index; WBC: white blood cells; IL: interleukin; W: women.

**Table 2:** Gene-specific DNA methylation and inflammatory markers.

Author	Study design	Outcome	Tissue type	Population Sex/Age/Sample size/ Country	Methylation sites/ method	Adjustment	Main findings	Clinical condition associated with the main findings^*∗*^
Piperi et al., 2010 [20]	CS	IL-6, IL-8, VEGF, COX-2	Tumour specimens	M and W/25-76/n=23/Greece	*MGMT, RARβ, RASSF1A, CDH13*/MS-PCR		IL-6: positive correlation with the four genes; IL-8 and COX-2: no correlation for any gene; VEGF: positive correlation with *MGMT* and *RARβ,* no correlation with RASSF1A and *CDH13*.	IPA: Cancer, neurological diseases, ophthalmic diseases.

Uddin et al., 2010 [27]	CS	IL-6, CRP	PBMC	M and W/ 45.3±16.7/ n=100/ USA	*IL-6*/Illumina HumanMethylation27K DNA Analysis BeadChip		Among patients with lifetime depression, there was a significant inverse correlation between methylation of *IL-6* and serum levels of IL-6 and CRP (Pearson r=-0.54, p=0.001 and Pearson r=-0.48, p=0.006, respectively.	*IL-6*: Rheumatic diseases, inflammatory bowel disease, Kaposi sarcoma.

Fu et al., 2011 [28]	CS	IL-10 (mRNA)	PBMC	M and W/39 ± 10.8/ n=40/ China	*IL-10 promoter*, 5 CpG sites/ Pyro Q-CpG system		Hypomethylation of -145C was correlated with higher IL-10 mRNA expression (r=-0.746, p=0.001). The authors did not report the results for the other CpG sites.	*IL-10*: Susceptibility to HIV type 1, rheumatic conditions, cutaneous leishmaniasis.

Zhang et al., 2012 [14]	CS	CRP	PB	M and W/18-78/ n=165/USA	*IL-6 promoter*, 6 sites/ bisulfite treatment	Age, sex, race, dietary folate intake, prudent diet pattern, western diet	With a spearman correlation coefficient of 0.11 (p=0.18), no association was found.	Not applicable

García-Cardona et al., 2014 [29]	CS	Leptin	PB	M and W/ 10-16/ n=106/ Mexico	*LEP promoter*/ MS-PCR		No significant correlation was observed between the circulating levels of leptin and the methylation frequencies of the two selected CpG sites of *LEP* promoter (at − 51 and − 31 nt).	Not applicable

Lai et al., 2014 [24]	CS	IL-6, TNF-*α* and CRP	PB	M and W/ 36-80/ n=46/China	*SOCS-1* gene, 11 CpG sites/ Bisulfite method		A positive trend between the levels of *SOCS-1* methylation and CRP levels was observed (R^2^=0.1127, p=0.0278). Patients with serum IL-6 above median showed a significantly higher *SOCS-1* methylation than the patients with serum IL-6 below median (p<0.001). Similar results were observed for TNF-*α* (p<0.001).	*SOCS-1*: Cancer, hepatic system diseases, ophthalmological diseases.

Smith et al., 2014 [12]	CS and prospective	sTNFR2, IL-6	PBMC	W/56.4 ± 9.4/ n=61/USA	*USP2, TMEM49, SMAD3, DTNB*, 8 CpG sites/ HumanMethylation 450 Bead Cheap		At baseline, lower methylation at each of the 8 CpG sites was significantly correlated with increased sTNFR2 and IL-6.	IPA: Gastrointestinal diseases, hepatic system diseases, cancer (like gynaecological cancer), dermatological diseases.

Wang et al., 2014 [22]	CS	Fibrinogen and CRP	PB	M and W/16.2 ±1.2/ n=703/ USA	*LY86 *gene, 6 CpG sites / HumanMethylation 27 BeadChip and ThumanMethylation 450 BeadChip from Illumina	Age, sex, race, BMI and batch	They performed a principal component analysis to combine the six CpG sites into one score. The score of these CpG sites was significantly associated with fibrinogen (partial r=0.145, p<0.001) and CRP (partial r=0.114, p=0.005).	*LY86*: Pelvic inflammation, pulmonary interstitial emphysema.

Wei et al., 2016 [21]	CS	CRP	WBC	M and W/ /n=673/China	*IL-6* promoter/EZ DNA Methylation Kit	Age and sex	Plasma CRP levels were significantly associated with *IL-6* promoter methylation (p=0.025). One interquartile range increase in plasma CRP was associated with a decrease in *IL-6* methylation by 0.78% (95% CI: -1.47% to -0.1%).	*IL-6*: Rheumatic diseases, inflammatory bowel disease, Kaposi sarcoma.

Arpón et al., 2017 [25]	CS	TNF-*α*, VCAM-1, sICAM-1, CRP, leptin	PB	M and W/63.8±2.74/ n=36/ Spain	*EEF2, COL18A1, IL4I1, LEPR, PLAGL1, IFRD1, MAPKAPK2 *and* PPARGC1B*/ llumina Infinium HumanMethylation 450K BeadChip		Results showed correlations between *LEPR* methylation and concentration of LEP (r=-0.24, p=0.047). Also, between *EEF2* methylation and concentration of TNF-*α* (r=0.24, p= 0.0408) and CRP (r=0.24, p=0.0457).	IPA: Inflammatory response, cardiovascular disease, reproductive system diseases.

Min A Jhun. et al., 2017 [23]	CS	CRP, IL-6, IL-18, fibrinogen	PBL	M and W/66 7.5/n=822/ USA	Cg0363183 in *F2RL3*/ Illumina Infinium HumanMethylation 27 BeadChips and the Illumina BeadXpress reader.	Age, sex, four principal component, five cell proportions, plate and random intercepts for family.	DNA methylation level of Cg03636183 in *F2RL3* was significantly associated with log (IL-18) levels (-0.11, 95% CI (-0.19, -0.04)).	Unknown.

Miller et al., 2017 [26]	CS	CRP	WBC	M and W/32.08±8.36/ n=286/ USA	*AIM2*,cg10636246/ llumina Infinium HumanMethylation 450K		Log CRP levels were negatively correlated with cg10636246 (r=-0.264, p<0.001).	*AIM2*: Skin disease, melanoma.

^*∗*^We used Ingenuity Pathway Analysis (IPA) for studies that found significant association between multiple inflammatory markers and/or methylation in multiple genes [12, 20, 25]. For the other studies, the connection between findings and disease was assessed through literature review and gene cards (https://www.genecards.org/).

CS: cross-sectional; IL: interleukin; VEGF: vascular endothelial growth factor; Cox-2: cyclooxygenase; M: men; W: women; MS-PCR: methylation-specific PCR; IPA: ingenuity pathway analysis; PBMC: peripheral blood mononuclear cells; HIV: human immunodeficiency virus; PB: peripheral blood; TNF- *α*: tumour necrosis factor-alpha; sTNFR2: soluble tumour necrosis factor receptor 2; BMI: body mass index; WBC: white blood cells; VCAM-1: vascular cell adhesion molecule 1; sICAM-1: soluble intercellular adhesion molecule 1; PBL: peripheral blood leucocytes.

**Table 3:** Epigenome-wide and histone acetylation approaches and inflammatory markers.

Author	Study design	Outcome	Tissue type	Population Sex/Age/Sample size/ Country	Methylation sites/ method	Adjustment	Main findings
*Epigenome-Wide Association Study*							

Guénard et al., 2013 [34]	CS	CRP	WB	M and W/12.25±5.77/ n=50/Canada	Infinium HumanMethylation 450K BeadChip	Age and sex	From 17 genes involved in the IL-8 signalling pathway, significant correlations between gene methylation and plasma CRP levels were found for 16 genes. Of these, 9 showed inverse correlation and 7 positive. Out of those 16 genes, 13 remained significant after adjustments.

Sun et al., 2013 [43]	CS	CPR	PBL	M and F/ 66.27±7.58/ n=966/ USA	Infinium HumanMethylation 27K BeadChip	Age, sex, BMI, smoking	207 out of 257 CRP-associated DNAm sites showed an inverse correlation of greater methylation with lower level of CRP. Twenty-four out of the top 30 CpGs remained significant in both replication subsets with *KLK10, LMO2 *and *TM4SF4* as top genes (p=5.85x10^−12^, p=1.69x10^−11^ and p=2.05x10^−10^, respectively).

Ligthart et al., 2016 [13]	CS	CRP	WB	M and W/mean age between 60 and 87/n=8,863/ Consortia	Illumina Infinium HumanMethylation 27K and 450K BeadChip.	Age, sex, white blood cell proportion, technical covariates, smoking, BMI.	218 CpG sites were significantly associated with CRP. Of those, 125 CpGs were positively associated and 93 were negatively associated. 58 CpG sites, in 47 genes, were still significantly associated in the replication cohort (n=4,111). The top CpG sites were located in *AIM, RPS6KA2 and PHOSPHO1* (P = 2.53x10^−27^, 2.06x10^−26^ and 4.87x10^−25^, respectively).

Marzi et al., 2016 [33]	CS	CRP	PB	M and W/60.9±8.89/ n=1741/ Germany	Illumina HumanMethylation 450K BeadChip	Age, sex, BMI, smoking, white blood cells composition.	Four CpG sites were significantly hypomethylated at high CRP concentrations. They were located at *AQP3, BCL3, SOCS3*, and intergenic at chromosome 19p13.2. Those four sites were replicated in three subcohorts: CpG at *AQP3* remained significant in two of the subcohorts and the one at *SOCS3* remained significant in one of the subcohorts.

Ahsan et al., 2017 [30]	CS	121 biomarkers related with inflammation, cancer, and cardiovascular disease.	PBL	M and W/ 14-97/ n=698/Sweden	Illumina HumanMethylation 450K BeadChip	Age, sex, batch and plate effects, year of sampling and cell fractions.	For 36% of the studied biomarkers (44/121), the abundance level was associated with DNA methylation, but for 52% of these biomarkers (23/44), the associations were explained by genetic variants. For a subset of biomarkers, the association with DNA methylation was confounded by environmental factors (e.g., smoking), but for the majority of the associations, no such relationship could be found.

Verschoor et al., 2017 (“The relation between…”) [31]	CS	TNF, IL-6, IL-8, IL-10	WB	M and W/48-78/n=14/Canada	Illumina Infinium HumanMethylation 450 K BeadChip	Age and sex	Serum IL-10 levels exhibited the most substantial association to DNA methylation patterns, followed by TNF, IL-6 and IL-8.

Verschoor et al., 2017 (“DNA methylation…”) [32]	CS	TNF, IL-6, IL-1*β*, IL-10 and CRP	PBMC	M and W/82-98/n=23/Canada	Illumina Infinium HumanMethylation 450 K BeadChip		Authors performed linear regression between each factor assessed and the scores of top 10 principal components (PCs) of the DNA methylation dataset. Only IL-6 and IL-10 were found to be associated, both of which with PC7 (ln IL-6, p = 0.002; ln IL-10, p= 0.03). ln CRP was positively associated with DNA methylation age using Hannum's approach (*β* = 0.21, p = 0.007), which relates to approximately 5-years age acceleration per 1-unit change in ln CRP (*β*= 0.20, p =0.008).

*Histone acetylation*							

da Silva et al., 2017 [15]	CS	IL-4, IL-6, IL-9, INF- *γ* and TGF-*β*	PBMC	M and W/ 68.5±6.49/n=10/ Brazil	Global Histone H4 Acetylation Assay Kit		At 24th session, the basal values of global histone H4 acetylation levels were correlated with basal IL-4 and IL-8 levels (r =−0.65, p= 0.04 and r=0.85, p=0.01, respectively).

CS: cross-sectional; WB: whole blood; M: men; W: women; IL: interleukin; PBL: peripheral blood leucocytes; BMI: body mass index; PB: peripheral blood; TNF: tumour necrosis factor; PBMC: peripheral blood mononuclear cells; INF-*γ*: interferon-gamma; TGF-*β*: transforming growth factor-beta.

## Supplementary Materials

Supplementary Materials Supplement Material S1: PRISMA guidelines checklist for reporting systematic reviews. Supplement Material S2: MOOSE guidelines checklist for reporting systematic reviews. Supplement Material S3: Search strategy. It provides a full description of the search strategies of all databases used in this systematic review. Supplement Material S4: Methods. It describes information about study selection and inclusion criteria, data extraction process, and risk of bias assessment. Supplement Table 1: Methylation of genes among candidate-gene approach studies.
