# Supplementary material for: Epigenetics and Inflammatory Markers: A Systematic Review of the Current Evidence
Source: Int J Inflam. 2019 May 8;2019:6273680. doi: 10.1155/2019/6273680 (PMC6530203; doi:10.1155/2019/6273680)
Supplement: Supplementary Materials — Supplement Material S1: PRISMA guidelines checklist for reporting systematic reviews. Supplement Material S2: MOOSE guidelines checklist for reporting systematic reviews. Supplement Material S3: Search strategy. It provides a full description of the search strategies of all databases used in this systematic review. Supplement Material S4: Methods. It describes information about study selection and inclusion criteria, data extraction process, and risk of bias assessment. Supplement Table 1: Methylation of genes among candidate-gene approach studies. [file 6273680.f1.docx]

**Supplement material**

**Supplement material S1.** PRISMA 2009 checklist

| **Section/topic** | **#** | **Checklist item** | **Reported on page #** |
| --- | --- | --- | --- |
| **TITLE** | | |  |
| Title | 1 | Identify the report as a systematic review, meta-analysis, or both. | 1 |
| **ABSTRACT** | | |  |
| Structured summary | 2 | Provide a structured summary including, as applicable: background; objectives; data sources; study eligibility criteria, participants, and interventions; study appraisal and synthesis methods; results; limitations; conclusions and implications of key findings; systematic review registration number. | 3 |
| **INTRODUCTION** | | |  |
| Rationale | 3 | Describe the rationale for the review in the context of what is already known. | 4 |
| Objectives | 4 | Provide an explicit statement of questions being addressed with reference to participants, interventions, comparisons, outcomes, and study design (PICOS). | 4 |
| **METHODS** | | |  |
| Protocol and registration | 5 | Indicate if a review protocol exists, if and where it can be accessed (e.g., Web address), and, if available, provide registration information including registration number. | 5 |
| Eligibility criteria | 6 | Specify study characteristics (e.g., PICOS, length of follow-up) and report characteristics (e.g., years considered, language, publication status) used as criteria for eligibility, giving rationale. | 5, Supplement Material 4 |
| Information sources | 7 | Describe all information sources (e.g., databases with dates of coverage, contact with study authors to identify additional studies) in the search and date last searched. | 5 |
| Search | 8 | Present full electronic search strategy for at least one database, including any limits used, such that it could be repeated. | 5, Supplement Material 3 |
| Study selection | 9 | State the process for selecting studies (i.e., screening, eligibility, included in systematic review, and, if applicable, included in the meta-analysis). | Supplement Material 4 |
| Data collection process | 10 | Describe method of data extraction from reports (e.g., piloted forms, independently, in duplicate) and any processes for obtaining and confirming data from investigators. | Supplement Material 4 |
| Data items | 11 | List and define all variables for which data were sought (e.g., PICOS, funding sources) and any assumptions and simplifications made. | Supplement Material 4 |
| Risk of bias in individual studies | 12 | Describe methods used for assessing risk of bias of individual studies (including specification of whether this was done at the study or outcome level), and how this information is to be used in any data synthesis. | Supplement Material 4 |
| Summary measures | 13 | State the principal summary measures (e.g., risk ratio, difference in means). | Supplement Material 4 |
| Synthesis of results | 14 | Describe the methods of handling data and combining results of studies, if done, including measures of consistency (e.g., I2) for each meta-analysis. | NA |
| Risk of bias across studies | 15 | Specify any assessment of risk of bias that may affect the cumulative evidence (e.g., publication bias, selective reporting within studies). | NA |
| Additional analyses | 16 | Describe methods of additional analyses (e.g., sensitivity or subgroup analyses, meta-regression), if done, indicating which were pre-specified. | NA |
| **RESULTS** | | |  |
| Study selection | 17 | Give numbers of studies screened, assessed for eligibility, and included in the review, with reasons for exclusions at each stage, ideally with a flow diagram. | Figure 1 |
| Study characteristics | 18 | For each study, present characteristics for which data were extracted (e.g., study size, PICOS, follow-up period) and provide the citations. | Table 1-3 |
| Risk of bias within studies | 19 | Present data on risk of bias of each study and, if available, any outcome level assessment (see item 12). | 6 |
| Results of individual studies | 20 | For all outcomes considered (benefits or harms), present, for each study: (a) simple summary data for each intervention group (b) effect estimates and confidence intervals, ideally with a forest plot. | 6-9, Table 1-3 |
| Synthesis of results | 21 | Present results of each meta-analysis done, including confidence intervals and measures of consistency. | NA |
| Risk of bias across studies | 22 | Present results of any assessment of risk of bias across studies (see item 15). | NA |
| Additional analysis | 23 | Give results of additional analyses, if done (e.g., sensitivity or subgroup analyses, meta-regression). | NA |
| **DISCUSSION** | | |  |
| Summary of evidence | 24 | Summarize the main findings including the strength of evidence for each main outcome; consider their relevance to key groups (e.g., healthcare providers, users, and policy makers). | 6-9, Table 1-3 |
| Limitations | 25 | Discuss limitations at study and outcome level (e.g., risk of bias), and at review-level (e.g., incomplete retrieval of identified research, reporting bias). | 12-14 |
| Conclusions | 26 | Provide a general interpretation of the results in the context of other evidence, and implications for future research. | 16 |
| **FUNDING** | | |  |
| Funding | 27 | Describe sources of funding for the systematic review and other support (e.g., supply of data); role of funders for the systematic review. | 17 |

*NA: not applicable

**Supplement material S2.** MOOSE checklist

| **Criteria** | | **Brief description of how the criteria were handled in the meta-analysis** |
| --- | --- | --- |
| **Reporting of background should include** | |  |
| √ | Problem definition | Epigenetic modifications of the genome, such as DNA methylation and histone modifications have been reported to play a role in the inflammation process. To date, however, little work has been done to systematically appraise the current evidence for the role of epigenetic markers on inflammation. |
| √ | Hypothesis statement | Epigenetic modifications (DNA methylation and histone modifications) are associated with inflammation. |
| √ | Description of study outcomes | Inflammatory markers |
| √ | Type of exposure or intervention used | Global DNA methylation, gene-specific DNA methylation and histone modifications. |
| √ | Type of study designs used | We included cross-sectional studies, case control studies and prospective studies. |
| √ | Study population | Alive humans |
| **Reporting of search strategy should include** | |  |
| √ | Qualifications of searchers | The credentials of the investigators are indicated in the authors list. |
| √ | Search strategy, including time period included in the synthesis and keywords | Search strategy and time periods are detailed in page 4 of the manuscript and in Appendix 3. |
| √ | Databases and registries searched | Embase.com, Medline (Ovid), Web-of-Science, Cochrane Central and Google Scholar. |
| √ | Search software used, name and version, including special features | We did not employ any search software. EndNote was used to merge retrieved citations and eliminate duplications. |
| √ | Use of hand searching | We hand-searched bibliographies of retrieved papers and relevant reviews for additional references. |
| √ | List of citations located and those excluded, including justifications | Details of the literature search process are outlined in the flow chart (Figure 1.). Citations for the included studies are enclosed in the table 1 and 2. The citation list for excluded studies is available upon request. |
| √ | Method of addressing articles published in languages other than English | We placed no restrictions on language. All identified studies were in English. |
| √ | Method of handling abstracts and unpublished studies | No unpublished studies were identified |
| √ | Description of any contact with authors | None |
| **Reporting of methods should include** | |  |
| √ | Description of relevance or appropriateness of studies assembled for assessing the hypothesis to be tested | Detailed inclusion and exclusion criteria are described in the Methods section. |
| √ | Rationale for the selection and coding of data | Data extracted from each of the studies were relevant to the population characteristics, study design, exposure, outcome, and possible effect modifiers of the association. |
| √ | Assessment of confounding | We assessed confounding by ranking individual studies based on different adjustment levels. |
| √ | Assessment of study quality, including blinding of quality assessors; stratification or regression on possible predictors of study results | Study quality was assessed using the Newcastle-Ottawa Scale, a semi-quantitative scale designed to evaluate the quality of nonrandomized studies. |
| √ | Assessment of heterogeneity | We did not perform any test of heterogeneity. |
| √ | Description of statistical methods in sufficient detail to be replicated | Not applicable |
| √ | Provision of appropriate tables and graphics | We included three tables. |
| **Reporting of results should include** | |  |
| √ | Graph summarizing individual study estimates and overall estimate | Not applicable |
| √ | Table giving descriptive information for each study included | Tables 1-3 |
| √ | Results of sensitivity testing | Not applicable |
| √ | Indication of statistical uncertainty of findings | When applied, 95% confidence intervals were presented with summary estimates. |
| **Reporting of discussion should include** | |  |
| √ | Quantitative assessment of bias | Newcastle-Ottawa scale was used to assess bias. |
| √ | Justification for exclusion | We excluded studies that used different exposure or outcome assessment for the comparison groups. |
| √ | Assessment of quality of included studies | We discussed the results of the Newcastle-Ottawa scale, and potential reasons for the observed heterogeneity. |
| **Reporting of conclusions should include** | |  |
| √ | Consideration of alternative explanations for observed results | We discussed that potential unmeasured confounders may have caused residual confounding. |
| √ | Generalization of the conclusions | We included data from more than 17,000 participants, including men and women from different ages. However, we noted that the majority of studies were from the USA. |
| √ | Guidelines for future research | We recommend future studies evaluate the impact of epigenetic marks in inflammation. |
| √ | Disclosure of funding source | No separate funding was necessary for the undertaking of this systematic review. |

**Supplement material S3.** Search strategy

**Embase.com**

(epigenetics/exp OR 'DNA methylation'/exp OR 'histone modification'/exp OR 's adenosylmethionine'/exp OR 'CpG island'/exp OR (((histone* OR dna OR 'long interspersed') NEAR/3 (acetylat* OR demethylat* OR methylat* OR phosphorylat* OR ubiquitinat* OR modif*)) OR 's adenosylmethionine' OR cpg OR epigenetic* OR epigenomic*):ab,ti) AND ( (inflammation/de AND (marker/de OR 'C reactive protein'/exp OR cytokine/de OR fibrinolysis/exp OR 'tumor necrosis factor alpha'/exp)) OR 'chronic inflammation'/exp OR ((inflammat* NEAR/3 (chronic* OR marker* OR biomarker* OR interleukin* OR crp OR 'c reactive' OR cytokine* OR fibrinolys* OR fibrinogenlys* OR 'tumor necrosis factor' OR tnf))):ab,ti) NOT ([animals]/lim NOT [humans]/lim) NOT ([Conference Abstract]/lim OR [Letter]/lim OR [Note]/lim OR [Editorial]/lim)

**Medline ovid**

(Epigenomics/ OR DNA methylation/ OR S-Adenosylmethionine/ OR CpG Islands/ OR (((histone* OR dna OR "long interspersed") ADJ3 (acetylat* OR demethylat* OR methylat* OR phosphorylat* OR ubiquitinat* OR modif*)) OR "s adenosylmethionine" OR cpg OR epigenetic* OR epigenomic*).ab,ti.) AND ( (inflammation/ AND (exp Biological Markers/ OR C-Reactive Protein/ OR cytokine/ OR fibrinolysis/ OR Tumor Necrosis Factor-alpha/)) OR ((inflammat* ADJ3 (chronic* OR marker* OR biomarker* OR interleukin* OR crp OR "c reactive" OR cytokine* OR fibrinolys* OR fibrinogenlys* OR "tumor necrosis factor" OR tnf))).ab,ti.) NOT (exp animals/ NOT humans/) NOT (letter OR news OR comment OR editorial OR congresses OR abstracts).pt.

**Cochrane central**

((((histone* OR dna OR 'long interspersed') NEAR/3 (acetylat* OR demethylat* OR methylat* OR phosphorylat* OR ubiquitinat* OR modif*)) OR 's adenosylmethionine' OR cpg OR epigenetic* OR epigenomic*):ab,ti) AND (((inflammat* NEAR/3 (chronic* OR marker* OR biomarker* OR interleukin* OR crp OR 'c reactive' OR cytokine* OR fibrinolys* OR fibrinogenlys* OR 'tumor necrosis factor' OR tnf)) OR atherosclero* OR ((intima-media OR intimamedia ) NEAR/3 thickness*) OR (coronar* NEAR/3 calcif*)):ab,ti)

**Web of science**

TS=(((((histone* OR dna OR "long interspersed") NEAR/2 (acetylat* OR demethylat* OR methylat* OR phosphorylat* OR ubiquitinat* OR modif*)) OR "s adenosylmethionine" OR cpg OR epigenetic* OR epigenomic*)) AND (((inflammat* NEAR/2 (chronic* OR marker* OR biomarker* OR interleukin* OR crp OR "c reactive" OR cytokine* OR fibrinolys* OR fibrinogenlys* OR "tumor necrosis factor" OR tnf)) OR atherosclero* OR ((intima-media OR intimamedia ) NEAR/2 thickness*) OR (coronar* NEAR/2 calcif*))) ) AND DT=(article)

**Google scholar**

"histone|dna methylation|modification"|epigenetics "chronic inflammation"|"inflammation marker|markers"

**Supplement material S4. Methods**

*Study selection and inclusion criteria:*

Studies were eligible for inclusion if they (i) were cross-sectional studies, case-control studies, case-cohort studies, nested case control studies or experimental trials; (ii) were conducted among humans; (iii) assessed epigenetic marks (global, site specific or genome-wide methylation of DNA or histone modifications); (iv) collected data on inflammatory markers (Complement system, angiotensin converting enzyme, coagulation system, C-reactive protein, TNF-α, leptin, interleukins, leukotrienes, histamine, prostaglandins or cytokines), and (v) reported the association of any of the upper mentioned epigenetic marks with inflammation markers. We did not make restriction on the tissue examined for epigenetic marks for inclusion. We excluded studies if they examined epigenetic marks other than DNA methylation and histone modifications, such as noncoding RNAs.

Two independent reviewers screened the retrieved titles and abstracts and selected eligible studies. If not consensus was reached, a third independent reviewer solved discrepancies between the two reviewers. Two reviewers retrieved full texts for studies that satisfied all selection criteria.

*Data extraction:*

A predesigned data collection form was prepared to extract the relevant information from the selected studies, including study design, characteristics of the study population, location of the study, age, confounders, and level of adjustment. Furthermore, for each study, the tissue type and methods used to determine DNA methylation, specific CpG sites, directions of the associations, and when possible the reported measures of associations (e.g., correlation analysis, beta-coefficients, odds ratio, relative risks, and confidence intervals) were reported.

*Assessing the risk of bias*

Two reviewers independently rated the quality of studies based on the Newcastle-Ottawa Scale (NOS), a semi-quantitative scale designed to evaluate the quality of case-control or cohort studies. We evaluated cross-sectional studies using an adapted version of the scale. The NOS assigns a maximum of four points for selection, two points for comparability, and three points for outcome. Studies that received a score of nine stars were judged to be at low risk of bias; studies that scored seven or eight stars were considered to be at medium risk and those that scored six or less were considered to be at high risk of bias.

*Outcome Assessment and Statistical Methods*

For each study, we defined whether an association was reported (e.g., correlation analysis, odds ratio, and relative risks), and when applicable, direction and effect sizes were reported. Heterogeneity permitting, we sought to pool the results using a random effects meta-analysis model. However, due to limited data, and differences in exposure and outcomes, and input parameters, it was not feasible to pool the data.

**Supplement Table 1.** Methylation of genes among candidate-gene approach studies.

| **Inflammatory marker** | **Tissue** | **Hypomethylated genes** | **Hypermethylated genes** | **Null association** | **Differentially methylated (unknown direction)** |
| --- | --- | --- | --- | --- | --- |
| IL-6 | Tumor specimens |  | *MGMT, RARβ, RASSF1A, CDH13* |  |  |
|  | PB |  | *SOCS-1* |  |  |
|  | PBMC | *IL-6, USP2, TMEM49, SMAD3, DTNB* |  |  |  |
| CPR | PBMC | *IL-6* |  |  |  |
|  | PB |  | *SOCS-1, LY86, EEF2* | *IL-6* promoter |  |
|  | WBC | *IL-6* promoter, *AIM2* |  |  |  |
| IL-8 | Tumor specimens |  |  | *MGMT, RARβ, RASSF1A, CDH13* |  |
| VEGF | Tumor specimens |  | *MGMT, RARβ* | *RASSF1A, CDH13* |  |
| Cox-2 | Tumor specimens |  |  | *MGMT, RARβ, RASSF1A, CDH13* |  |
| IL-10 | PBMC |  |  |  | *IL-10* promoter |
| Serum leptin | PB | *LEPR* |  | *LEP* promoter |  |
| TNF-α | PB |  | *SOCS-1, EEF2* |  |  |
| sTNFR2 | PBMC | *USP2, TMEM49, SMAD3, DTNB* |  |  |  |
| IL-18 | PBL | *F2RL3* |  |  |  |
| Fibrinogen | PBL |  | *LY86* |  |  |

PB: peripheral blood; PBMC: peripheral blood mononuclear cell; WBC: white blood cell; PBL: Peripheral blood lymphocytes
